# Cryogenic electron tomography reveals novel structures in the apical complex of *Plasmodium falciparum*

**DOI:** 10.1128/mbio.02864-23

**Published:** 2024-03-08

**Authors:** Stella Y. Sun, Li-av Segev-Zarko, Grigore D. Pintilie, Chi Yong Kim, Sophia R. Staggers, Michael F. Schmid, Elizabeth S. Egan, Wah Chiu, John C. Boothroyd

**Affiliations:** 1 Department of Structural Biology, University of Pittsburgh, Pittsburgh, Pennsylvania, USA; 2 Department of Bioengineering, James H. Clark Center, Stanford University, Stanford, California, USA; 3 Department of Microbiology and Immunology, Stanford University School of Medicine, Stanford, California, USA; 4 Department of Pediatrics, Stanford University School of Medicine, Stanford, California, USA; 5 Division of Cryo-EM and Bioimaging, SSRL, SLAC National Accelerator Laboratory, Stanford University, Menlo Park, California, USA; The George Washington University Milken Institute of Public Health, Washington, DC, USA

**Keywords:** cryo-electron tomography, subtomogram averaging, rhoptry, apical ring, *Plasmodium falciparum*, *Toxoplasma gondii*

## Abstract

Intracellular infectious agents, like the malaria parasite, *Plasmodium falciparum*, face the daunting challenge of how to invade a host cell. This problem may be even harder when the host cell in question is the enucleated red blood cell, which lacks the host machinery co-opted by many pathogens for internalization. Evolution has provided *P. falciparum* and related single-celled parasites within the phylum Apicomplexa with a collection of organelles at their apical end that mediate invasion. This apical complex includes at least two sets of secretory organelles, micronemes and rhoptries, and several structural features like apical rings and a putative pore through which proteins may be introduced into the host cell during invasion. We perform cryogenic electron tomography (cryo-ET) equipped with Volta Phase Plate on isolated and vitrified merozoites to visualize the apical machinery. Through tomographic reconstruction of cellular compartments, we see new details of known structures like the rhoptry tip interacting directly with a rosette resembling the recently described rhoptry secretory apparatus (RSA)*,* or with an apical vesicle docked beneath the RSA. Subtomogram averaging reveals that the apical rings have a fixed number of repeating units, each of which is similar in overall size and shape to the units in the apical rings of tachyzoites of *Toxoplasma gondii*. Comparison of these polar rings in *Plasmodium* and *Toxoplasma* parasites also reveals them to have a structurally conserved assembly pattern. These results provide new insight into the essential and structurally conserved features of this remarkable machinery used by apicomplexan parasites to invade their respective host cells.

**IMPORTANCE:**

Malaria is an infectious disease caused by parasites of the genus *Plasmodium* and is a leading cause of morbidity and mortality globally. Upon infection, *Plasmodium* parasites invade and replicate in red blood cells, where they are largely protected from the immune system. To enter host cells, the parasites employ a specialized apparatus at their anterior end. In this study, advanced imaging techniques like cryogenic electron tomography (cryo-ET) and Volta Phase Plate enable unprecedented visualization of whole *Plasmodium falciparum* merozoites, revealing previously unknown structural details of their invasion machinery. Key findings include new insights into the structural conservation of apical rings shared between *Plasmodium* and its apicomplexan cousin, *Toxoplasma*. These discoveries shed light on the essential and conserved elements of the invasion machinery used by these pathogens. Moreover, the research provides a foundation for understanding the molecular mechanisms underlying parasite-host interactions, potentially informing strategies for combating diseases caused by apicomplexan parasites.

## INTRODUCTION

*Plasmodium falciparum* (*P. falciparum*) is the causative agent of human malaria and, as such, represents a pathogen of global importance. Despite enormous efforts to control it, this apicomplexan parasite continues to infect >220 million people each year, resulting in the deaths of ~627,000 individuals per year, mostly children under the age of five (1). One of the challenges in controlling this ongoing pandemic is the rapidity with which drug-resistance emerges, making the discovery of new drugs and new drug targets an urgent task.

Like other Apicomplexa, *P. falciparum* is an obligate intracellular, single-celled parasite. Infections begin with introduction of sporozoites through the bite of an infected mosquito. These migrate to the liver where they infect hepatocytes, yielding several thousand infectious merozoites that are released into the bloodstream and go on to infect mature red blood cells (RBCs). After RBC invasion, the internalized merozoite undergoes a well-defined developmental cycle in which first the ring stage, then trophozoites, and finally schizonts are produced over ~48 hours, culminating in the release of upwards of 30 daughter merozoites from each infected RBC (2). These, in turn, can go on to infect additional RBCs, producing waves of parasitemia. In this study, we focus on the merozoites, the small, invasive stage that transits between RBCs, for direct cryogenic electron tomography (cryo-ET) imaging without sectioning or milling, which is usually required for thick cellular samples >1 μm (3).

Invasion by merozoites is an active process driven mostly by the parasites themselves (4–6). The first step is attachment through proteins present on the parasites’ surface binding to molecules on the RBC surface. This interaction can occur seemingly at almost any portion of the parasite’s and host’s respective surfaces. There soon follows, however, a reorientation of the parasite such that its apical end, containing key invasion machinery, is positioned in contact with the RBC. Next, the bulb-shaped secretory organelles, known as rhoptries, inject the contents of their apical necks into the host cell. These rhoptry neck proteins, or “RONs,” interact with host cytoskeletal components, providing a key part of the machinery necessary for invasion. One RON protein, PfRON2, integrates into the RBC plasma membrane, forming a bridge between the RBC surface and its underlying cytoskeleton (7). PfRON2 binds a protein on the merozoite’s surface, PfAMA1, which is also an integral membrane protein, in this case originating from a different set of secretory organelles known as micronemes; these deposit their protein contents onto the merozoite’s surface through a mechanism whose details are not well understood, but presumed to involve fusion of micronemes with the parasite’s plasma membrane. Once PfRON2 and PfAMA1 have bound one another, internalization can commence.

The rhoptries are secretory, bulb-shaped organelles that in *Plasmodium* are present as a pair, with their tapered ends oriented toward the merozoite’s apical end. The two rhoptries can also be found to be fused at their necks or go through complete fusion with each other during invasion (8–10). Their contents, which include many important effectors in addition to the RON proteins described above, are electron dense by transmission electron microscopy using conventional staining and fixation methods. Micronemes, which are also generally apical in placement, are much smaller than rhoptries and enriched at the apical region around the rhoptry. In addition to these two secretory organelles, the apical complex for which the phylum is named includes cytoskeleton elements, including a set of anterior rings for which the precise number depends on the species and the imaging method used (11). The most basal of these in *Toxoplasma* tachyzoites is the “apical polar ring” (APR), which is attached to the subpellicular microtubules (SPMTs) and is therefore thought to function as a microtubule-organizing center (12–14). The ring-like structures that have been described apical to the APR have been referred to in *P. falciparum* as apical rings (9, 12), in *Plasmodium berghei* as conoidal rings (12), or in *Toxoplasma gondii* (*T. gondii*) and *Cryptosporidium parvum* (*C. parvum*) as preconoidal rings (13, 15). Given that in the *Plasmodium* merozoites there is no conoid cage and intraconoidal microtubules, we will refer to them here simply as “apical rings”. While this paper was in preparation, related findings were reported by Martinez et al. (15), Dos Santos et al. (16), and Lopez et al. (17), showing that the preconoidal rings serve as a hub for factors associated with motility and invasion. Eight components have been found to localize above the conoid in both *T. gondii* and *C. parvum* (16, 17); however, only three of them have orthologs in *Plasmodium*. The precise composition of these apical rings and their role in invasion or other biological functions of the *Plasmodium* merozoite are not yet known.

To help shed light on the interactions and possible functions of all these apical components, we have applied recently developed methods of cryo-ET to study merozoites embedded in vitreous ice without chemical fixation and staining. We report here the results of this examination, including details on the rhoptries and additional membrane-bound organelles localized at the apical complex and new insight into the complexity of the apical ring structures.

## RESULTS

### Cryo-ET of isolated merozoites reveals new details of the apical complex

**Fig 1:**
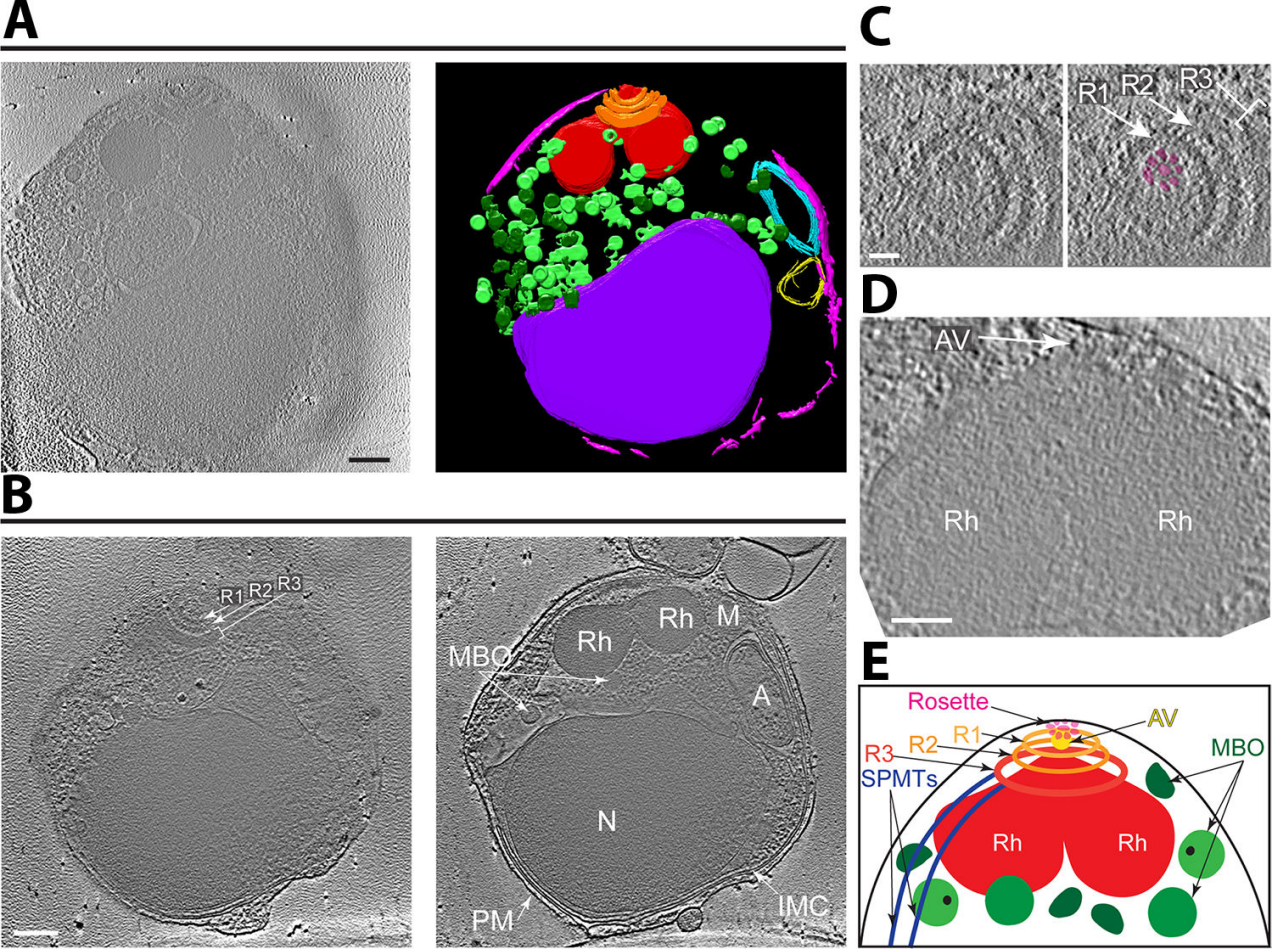
Cryo-ET with Volta Phase Plate reveals the organization of subcellular organelles in *Plasmodium falciparum* merozoites. (**A**) Tomographic slice of a representative merozoite (left) and its 3D annotation showing the apical rings (orange), rhoptries (red), membrane-bound organelles (light/dark green), inner membrane complex (pink), apicoplast (cyan), mitochondrion (yellow, presumptively designated based on double-membrane structure and position adjacent to the four-membrane apicoplast), and nucleus (purple). Scale bar, 200 nm. Movie with the complete tomogram and annotation is in Video S1. (**B**) Two tomographic slices of a merozoite (different from that shown in [**A**]) that better show the three apical rings (Ring 1 [R1], Ring 2 [R2], Ring 3 [R3]), rhoptries (Rh), membrane-bound organelles (MBO), inner membrane complex (IMC), apicoplast (A), mitochondrion (M), nucleus (N), and plasma membrane (PM). Scale bar, 200 nm. (**C**) Zoomed-in view of a tomographic slice of a merozoite (different from [**A**] and [**B**]) showing the apical rings (Rings 1 and 2, arrows; Ring 3, bracket) and the rosette (highlighted in magenta). Scale bar, 50 nm. (**D**) Zoomed-in view of a tomographic slice of a merozoite (different from [**A**], [**B**], and [**C**]) showing the apical vesicle (AV; arrow) at the tip of two rhoptries (R). Scale bar, 100 nm. (**E**) Cartoon of the *Plasmodium* merozoite apical complex showing the arrangement of key subcellular structures. Abbreviations as in (**B**) except subpellicular microtubules (SPMTs) and apical vesicle (AV) are also indicated.

*P. falciparum* (strain 3D7) was grown in human blood using established methods and synchronized using successive rounds of sorbitol and magnetic purification. Cultures at the early schizont stage were incubated with the protease inhibitor E-64 for 5–6 hours, and then free merozoites were harvested using syringe filtration as previously described (18). After washing the harvested merozoites, they were concentrated and loaded onto a Lacey carbon grid, followed by one-side blotting and plunge-freezing in liquid ethane. This freezing process avoids artifacts caused by chemical fixation or staining used for conventional transmission electron microscope sample preparation. The frozen grids were transferred to a 200-kV Talos Arctica electron microscope and imaged with Volta Phase Plate optics and sample-stage tilting. Each tilt image followed a step of 2- or 3-degree increments in order to compute a 3D volume of the sample. A representative tomogram of the whole merozoite cell is presented in Video S1. Virtual sections from this tomogram and other representative tomograms are presented in Fig. 1.

Given the limited penetrating capability of an electron beam, cryo-ET is generally restricted to cellular samples <0.5 µm. We were therefore surprised that our tomogram captured not only the tapering apical end, which was our primary objective, but also the entire length and width of many parasites that normally are roughly spherical and about 1.5 µm in diameter. We attribute this to the parasites being disrupted during sample preparation and cannot exclude the possibility that there was consequent loss of some of their contents. Nevertheless, we readily identified all the known cytoskeletal structures and organelles of the merozoite, including their apical rings with associated SPMTs, a pair of rhoptries, membrane-bound organelles that presumably are a mix of micronemes and dense granules, the inner membrane complex (IMC), the nucleus, the apicoplast with its multiple delimiting membranes, and mitochondria (Fig. 1). Our primary interest is in the components of the apical complex, which we and others have recently analyzed in detail in cryo-ET studies with the related apicomplexan parasite, *T. gondii* (19–21); we therefore focused our attention on this area. For each of the quantifications presented in this work, we used only those tomograms that identified the relevant feature(s). Of particular note was the presence of a distinct rosette of particles at the extreme apical tip of the merozoites (Fig. 1C). This and other features are described in more detail below.

### Apparent densities associate with the membrane-bound organelles

**Fig 2:**
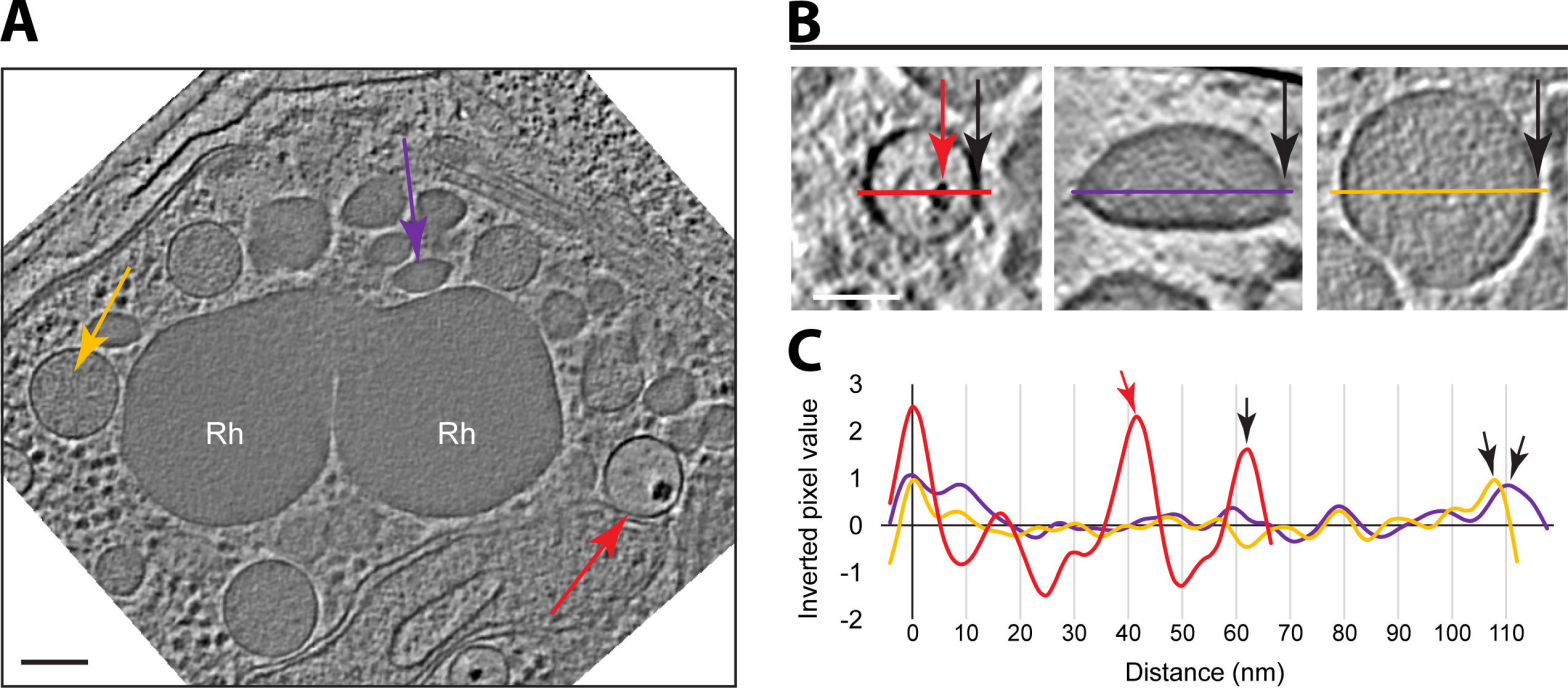
Three distinct subclasses of membrane-bound organelles are present. (**A**) A tomographic slice of a *Plasmodium* merozoite showing the rhoptries (Rh), and three distinct subclasses of membrane-bound organelles, MBO1, MBO2, and MBO3, marked with yellow, purple, and red arrows, respectively. Scale bar, 100 nm. (**B**) Zoomed-in views of a representative of each subclass, including a line colored as in (**A**) and with a black arrow marking the right edge in all cases. The red arrow indicates the electron-dense region characteristic of MBO3s. Scale bar, 50 nm. (**C**) Inverted pixel values of the lines shown in (**B**). Arrows also as in (**B**).

In cells that appeared minimally disrupted, we identified, in addition to the rhoptries, three subclasses of membrane-bound organelles (MBO), distinguished by their shape and small size (Fig. 2). Although the contents of two subclasses appeared relatively homogeneous (Fig. 2A yellow and purple arrows), the third subclass of MBO had a very electron-dense cluster inside (Fig. 2A red arrow, and Fig. 2B). Which, if any, of these subclasses corresponds to the micronemes or dense granules cannot be discerned from the data presented here; however, due to their distinct shapes and densities, they seem likely to have correspondingly distinct cargoes, and thus, functions.

Close inspection of the rhoptry interior mostly revealed a granulated texture with no apparent subsections or ordered densities. Along the inner side of the rhoptry membrane, however, we identified an additional density resembling a lining (Fig. S1A). On the outer surface of this organelle, we observed scattered, discrete globular densities attached to only the neck area of the rhoptries, while the rest of the exterior surface of the rhoptry bulb appeared smoother (Fig. S1B through E). The identity and significance of these densities are not known.

In addition to the single-membrane-bound organelles described above, we also observed double- and quadruple-membrane-bound organelles located adjacent to one another (Fig. 1; Fig. S2); these have all the very characteristic features, and are therefore presumed to be, the mitochondrion and apicoplast, respectively (22, 23). When both structures were detected in the same tomographic slice, they were always observed next to one another, as previously reported (24, 25) (Fig. S2). The shortest distance between the outer membrane of the mitochondrion and the apicoplast was ~14 nm, similar to the distance between the inner and outer membranes of the mitochondria or the two outermost membranes of the apicoplast in the same area (Fig. S2B). A close inspection revealed densities in the interface between the mitochondria and the apicoplast in the areas of intimate association, suggesting a discrete structure connects them (Fig S2C and D).

### A rosette is apparent on the merozoite surface with an apical vesicle beneath it

**Fig 3:**
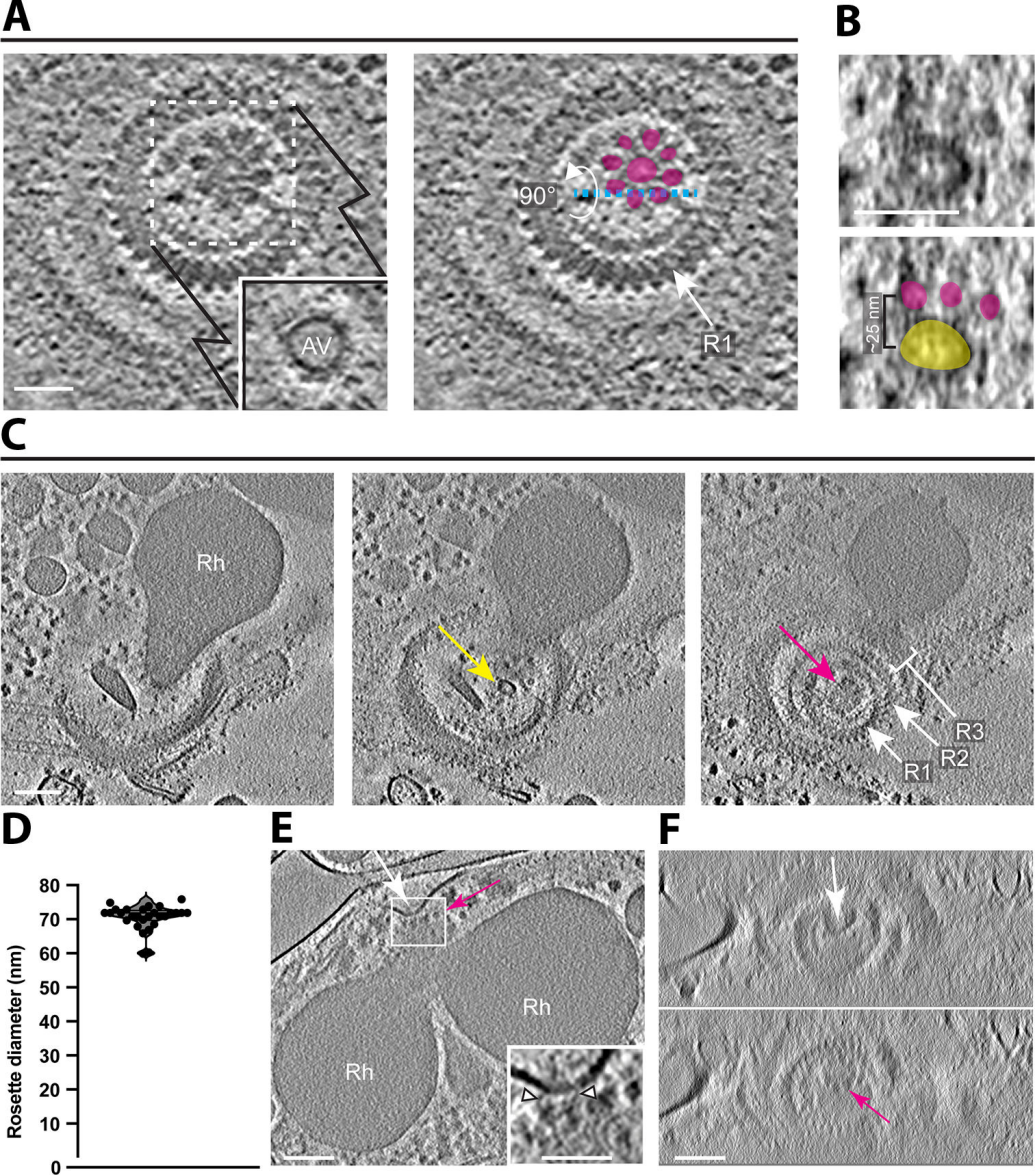
Rosettes are associated with either an apical vesicle (AV) or a rhoptry tip. (**A**) A tomographic slice showing a top view of Ring 1 (R1) surrounding a rosette. The inset shows an AV in a z-slice just below the area indicated by the dashed-line square. The right panel highlights the densities of the rosette in magenta and an arrow to point to R1. Scale bar, 50 nm. (**B**) A zoomed-in side view of the rosette and the AV from (**A**) showing the orthogonal sectioning plane labeled in cyan dashed line. The bottom panel highlights the rosette in magenta and the AV in yellow. (**C**) Three tomographic slices from a single tomogram (different from [**A**]) showing a single rhoptry (Rh) with an AV at its tip (yellow arrow) and above it a rosette (magenta arrow) surrounded by the three apical rings (R1, R2, R3). Scale bar, 100 nm. (**D**) Rosette diameters (between outer edges) as measured from the top view of the rosette (mean ± SD = 70.7 ± 3.7 nm, *N* = 28). (**E**) A tomographic slice showing a dimple in the parasite membrane (white arrow) in the area of association with the rosette (seen from a side view, magenta arrow) and the two rhoptries (Rh). Scale bar, 100 nm. A zoomed-in view of the rectangle shows densities between the parasite membrane and the rosette (arrow heads). Scale bar, 50 nm. (**F**) An orthogonal view of the tomogram in (**E**) showing the top-down view of the dimple in the parasite membrane (top panel, white arrow) and the rosette in the z-slices just below (bottom panel, magenta arrow). Images are a stack of seven consecutive slices. Scale bar, 100 nm.

We observed a distinct density pattern at the apical end of these merozoites, consisting of a rosette embedded within the parasite’s plasma membrane and concentric with the apical rings (Fig. 3; Video S2). Each rosette has an eightfold rotational symmetry with an overall diameter of ~70 nm and also has an axial ring-like density (Fig. 3A). A similar rosette-like structure has been described at the apical end of *Toxoplasma* tachyzoites, *Cryptosporidium* sporozoites, and *Plasmodium* merozoites, and has been termed the rhoptry secretory apparatus (RSA) because of its presumed role in secretion by these organelles (19). In 14 out of 39 tomograms, we identified an apical vesicle immediately beneath the rosette (Fig. 3A; Video S2). The shape of the apical vesicles was often less spherical than the apical vesicles observed in *Toxoplasma* with an average diameter of 46.6 ± 4.2 nm (*N* = 14). In instances where the vesicle was missing, the tip of the rhoptries appeared to directly interact with the rosette (Fig. 3E and F; Fig. S3; Video S3). This interaction was evident even in cases where the plasma membrane integrity was compromised, and the rhoptries were separated from their position close to the apical rings. This condition resulted in an elongated structure that appeared to be membrane-limited and that extended all the way up into and through the space surrounded by the apical rings, eventually reaching the merozoite surface (Fig. S3; Video S3). Although the origin of this membrane cannot be definitively discerned from these images, the data are consistent with it being an extended invagination of the parasite’s plasma membrane, perhaps a result of the rhoptries being firmly attached to the membrane (through the RSA) and pulled away during sample preparation.

### The apical rings present a distinctive structure containing 34 repeating units

**Fig 4:**
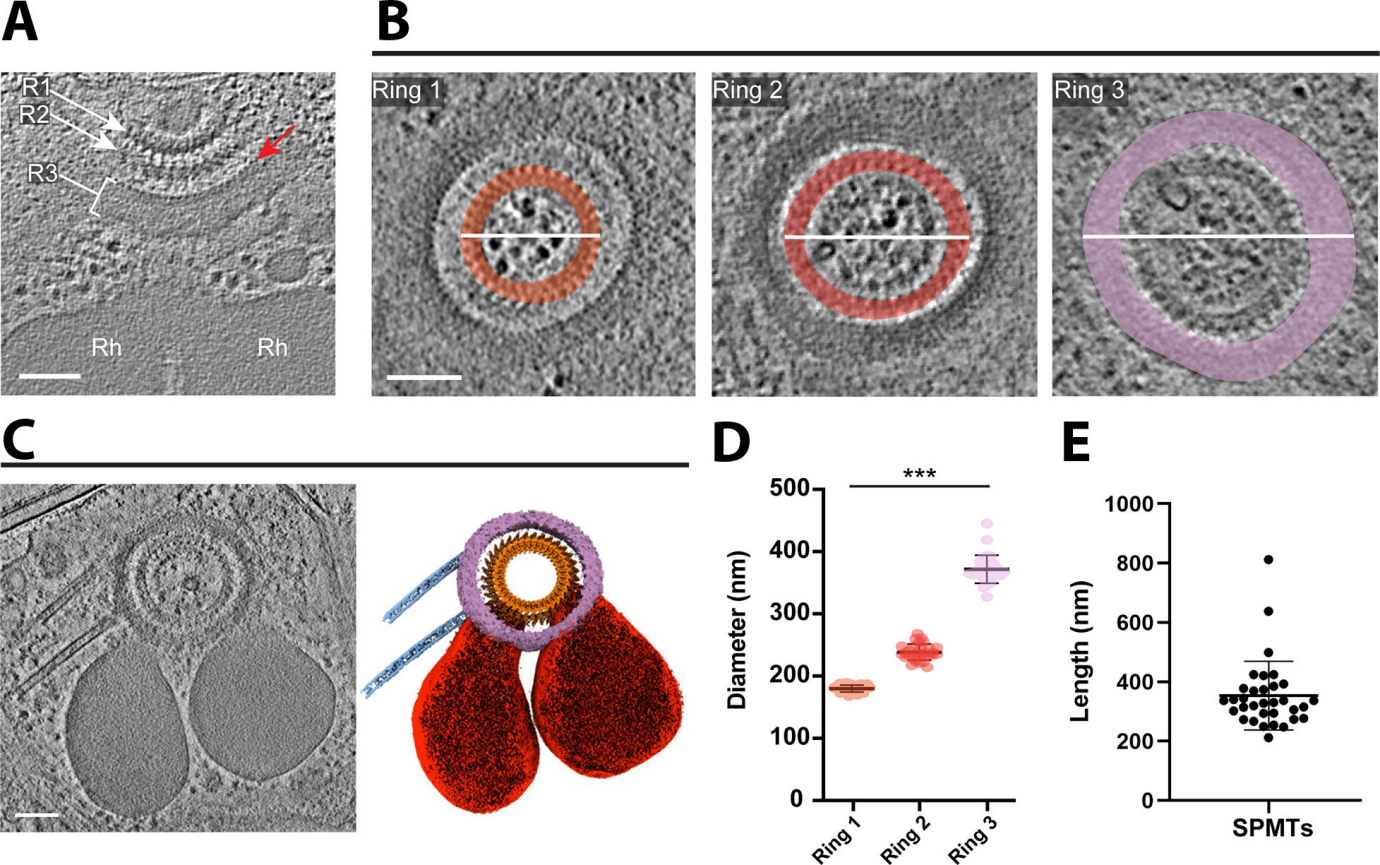
The apical rings appear as three distinct entities. (**A**) A tomographic slice showing the rhoptries (Rh) and three apical rings (R1, R2, R3). The red arrow points to “bead-like” densities on the innermost edge of Ring 3. Scale bar, 500 nm. (**B**) Three tomographic slices from a tomogram (different from [**A**]) showing a top view of the three apical rings, highlighted in different colors (orange, red, and violet for Rings 1, 2, and 3, respectively). The white lines represent the diameter that was measured for (**D**). Scale bar, 100 nm. (**C**) The left panel is a tomographic slice showing the rhoptries, three apical rings, the apical vesicle, and two SPMTs. The right panel is the cryo-ET average densities of the three apical rings (orange and purple) and the annotation of SPMTs (blue) and rhoptries (red). Scale bar, 100 nm. (**D**) The mean maximum diameter for each apical ring. Ring 1 mean ± SD = 180 ± 6 nm, *N* = 30; Ring 2 mean ± SD = 238 ± 13 nm, *N* = 30; Ring 3 mean ± SD = 372 ± 23 nm, *N* = 29. (**E**) The mean length of associated SPMTs is 354 ± 116 nm, *N* = 33.

**Fig 5:**
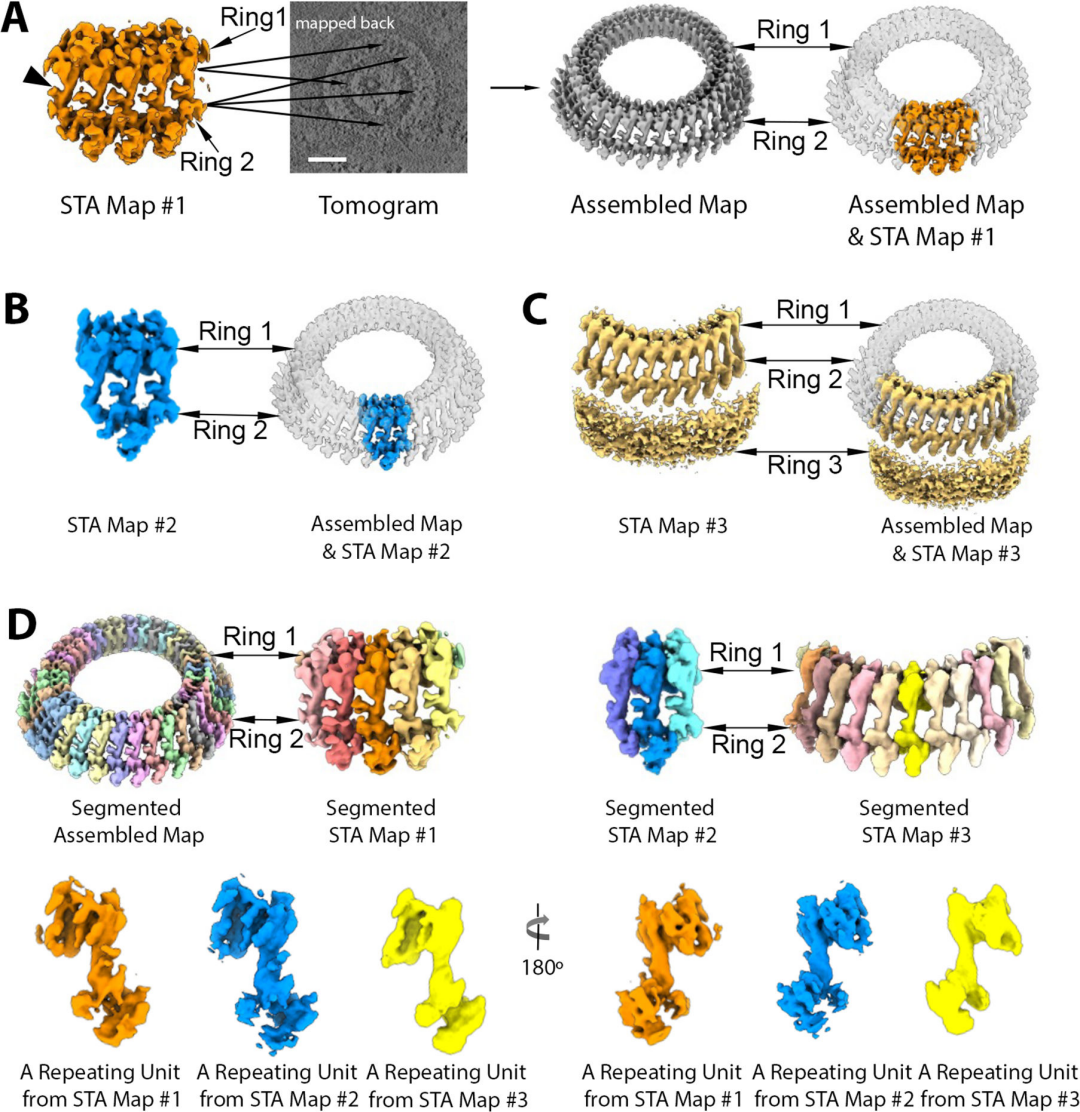
Rings 1 and 2 of *Plasmodium falciparum* merozoites contain 34 repeating units. Three subtomogram average (STA) maps of *Plasmodium* apical rings were generated and shown in A, B, and C. (**A**) STA map #1 (orange) containing five “bridge” stalks (arrowhead) connecting Ring 1 to Ring 2 was mapped back and aligned into a tomogram at multiple positions to produce the assembled map (grey). (**B**) STA map #2 (blue) containing three bridge stalks was generated by focus refinement based on the middle bridge stalk and aligned into the assembled map. (**C**) STA map #3 (yellow) was generated by focus refinement based on Ring 2, showing high densities of Ring 3 surrounding Rings 1 and 2. (**D**) The assembled map and three STA maps were segmented into repeating units shown in multiple random colors. A single repeating unit from each STA map is presented in two orientations, differing by 180°. Each color represents a repeating unit from each STA map as indicated in the figure. More detailed density features were observed in the STA maps #1 and #2.

Three generally distinct, concentric rings were apparent at the apical end of the parasites, with diameters of ~180, ~240, and ~370 nm shown in slice view, respectively (Fig. 4A and B). To gain structural details on the smallest, most apical ring (“Ring 1”) as well as the next closest ring (“Ring 2”) and the apparent “bridge” density connecting the two, we performed subtomogram averaging on extracted particles, each containing five repeating bridge densities. The subtomogram averaging (STA) map #1 was generated at ~41 Å and mapped back to the cellular tomograms to assemble the complete Rings 1 and 2 (Fig. 5A).

To determine the precise number of repeating units from the top view, focus refinement was further applied to Rings 1 and 2 based on only one bridge density to reveal more features of structural densities for each repeating unit (Fig. 5B). STA map #2 was generated by focused refinement and fitted into the assembled map of the whole ring structure (Fig. 5B). Based on the vertical repeating pattern of bridge densities, a small, distinct unit spanning both rings can be further segmented into each STA map and the entire structure (Fig. 5D).

Based on the density volume and using prior calibration to the tubulin subunits of microtubules in *Toxoplasma*, the total mass of the repeating unit of this ring complex can be estimated to be between 13.6 and 16.3 MDa. The assembled map of the whole Ring 1 and 2 contains 34 highly reproducible units based on the horizontal slice view and 3D volume of the cryo-ET density map from 27 out of 39 tomograms, indicating this is the number of units comprising both rings (Fig. 5D; Fig. S4; Video S4). Although fewer repeating units were observed in some tomograms, the difference appeared due to the partial or off-angle view that did not allow a complete image of the entire ring; in no case were more than 34 repeating units observed. In some cases, the rings appeared bent into somewhat distorted circles, suggesting some flexibility in the connection between each ring unit (Fig. S5). Ring 1 can be further segmented into an inner ring (pink) with a diameter of ~125.6 nm and an outer ring density (red) with a diameter of ~186.8 nm (Fig. S4B and S6C). The spacing between the neighboring units of outer Ring 1 was ~17.3 nm (Fig. S6B). The segmentation of Ring 2 revealed a circular density of the inner ring (blue) associated with 34 repeating densities (green) with a diameter of ~232 nm and a ~21.7 nm neighbor spacing (Fig. S4C, S6B and C). Most importantly, Rings 1 and 2 can be seen to be clearly connected via a “bridge” (Fig. S4A, yellow), which also presents as 34 repeating units, connecting the bottom, outer edge of Ring 1, and the inner, upper edge of Ring 2. Thus, these two rings are clearly connected.

Using Ring 2 and the bridge portion as anchor points, focus refinement was applied to Ring 3, resulting in STA map #3 that appeared relatively amorphous, had no obvious repeating features, and was about 50-nm thick (Fig. 5C). The resulting cryo-ET density map fitted into the assembled map of the whole ring, presenting the space correlation between the Rings 1, 2, and 3. The results revealed distinct bead-like densities visible on the inner most edge of Ring 3 (Fig. 4A), but no repeating mass was resolved in the subtomogram averaged map (Fig. 5C). Either one or two subpellicular microtubules were anchored to the edge of Ring 3 in 21 tomograms (*N* = 39) with a length ranging between 212 and 812 nm for any one microtubule (mean ± SD = 354 ± 116 nm, *N* = 33) (Fig. 4C and E).

**Fig 6:**
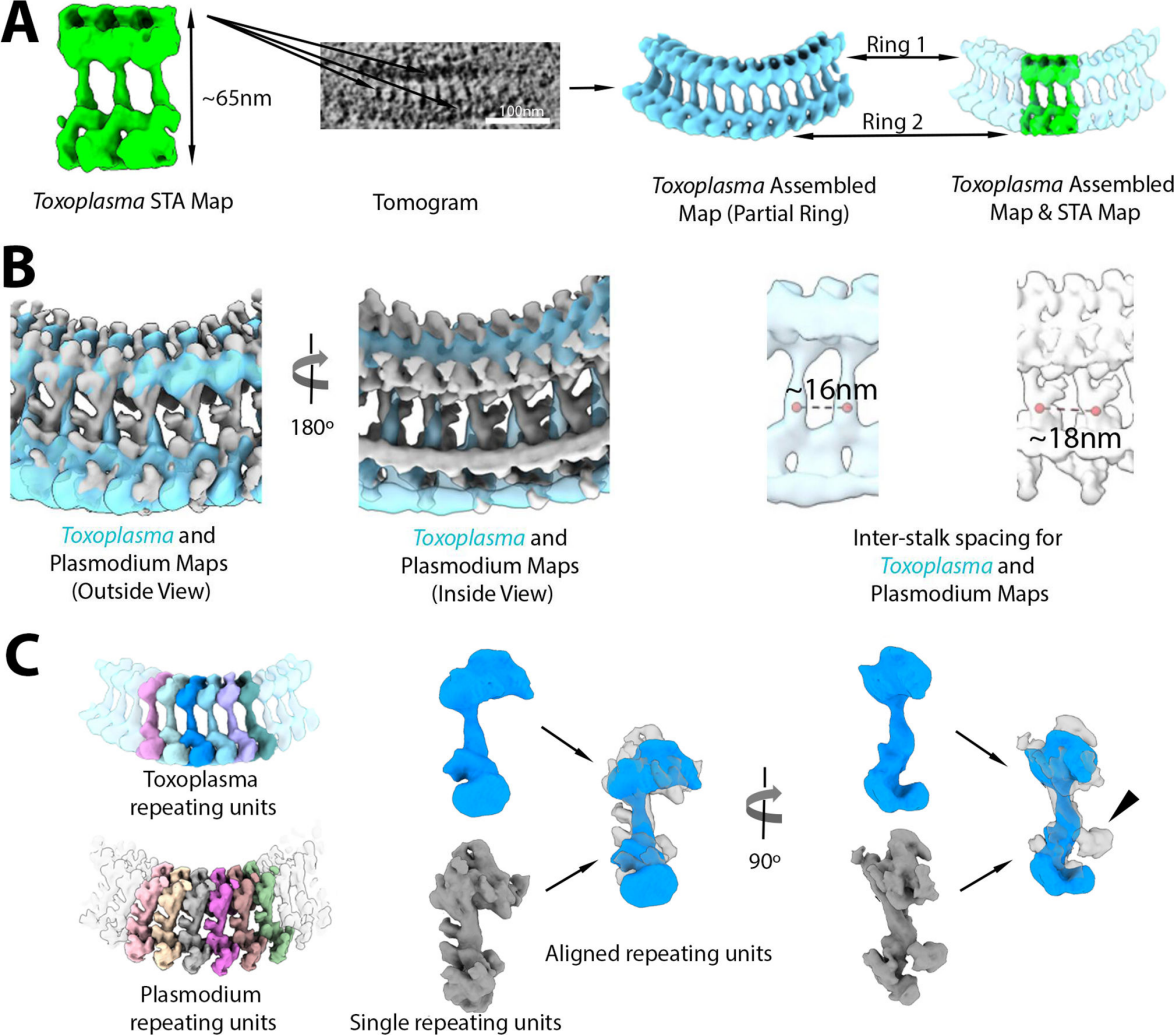
*Plasmodium falciparum* Rings 1 and 2 show a similar repeating unit and pattern to those seen for the pre-conoidal rings of *Toxoplasma gondii*. (**A**) A subtomogram average map of the *Toxoplasma* apical rings (green) containing three bridge stalks was generated and mapped back to a tomogram, producing an assembled map (cyan blue). (**B**) The assembled maps of *Toxoplasma* (cyan blue) and *Plasmodium* (gray) viewed from inside and outside the apical rings are superimposed to show similar geometries overall. Note that the spacing of the bridge stalks differs, with ~16 nm for *Toxoplasma* and ~18 nm for *Plasmodium*. (**C**) The repeating units (random colors) in the two assembled maps were segmented in *Toxoplasma* like in *Plasmodium*. A single unit from *Toxoplasma* (blue) and *Plasmodium* (gray) was superimposed for comparison. Extra density of the bridge stalk can be seen for *Plasmodium* relative to *Toxoplasma* (arrowhead).

The subtomogram averaged map was then used to compare the overall density of Rings 1 and 2 of *Plasmodium* merozoites with that of the repeating units averaged from the preconoidal rings of *Toxoplasma* tachyzoites at ~60 Å (Fig. 6; Video S5). The overall dimension of the *Toxoplasma* preconoidal rings is ~220 nm in maximum outer diameter shown in slice view and their overall arrangement was similar to what we observed for *Plasmodium* in being two concentric and somewhat discrete rings with a bridge linking the two (Fig. 6A). When the rings of the two species were aligned, the fitting result showed substantial similarities in overall shape, although the bridge densities appeared to have slightly different tilts (Fig. 6B). In addition, when aligned, the bridge stalk of the preconoidal ring of *Plasmodium* has an extra density protruded that is not observed in *Toxoplasma* (Fig. 6B and C). The space between two successive densities bridging the two rings is about ~16 and ~18 nm for *Plasmodium* and *Toxoplasma*, respectively (Fig. 6B). In addition, the *Toxoplasma* repeating units were considerably more in number; although we were not able to determine a precise count, we observed 42–45 units in five tomograms where they could be estimated. Importantly, the overall size and shape of the repeating units segmented from Rings 1 and 2 of *Toxoplasma* and *Plasmodium* are similar (Fig. 6C).

## DISCUSSION

We report here tomographic images of vitrified *P. falciparum* merozoites, revealing intriguing structures at their apical end. One of the structures observed is a rosette, which extends from the tip of a rhoptry neck or apical vesicle adjacent to just below the parasite membrane. Given their size, pattern, and position, and as recently reported by others (10), these appear analogous, and likely homologous, to the RSA described in the related *Toxoplasma* and *Cryptosporidium* (19). As shown by those authors, this structure appears to have a common evolutionary origin with the discharge apparatus of the very distantly related ciliates like *Paramecium*. As for the Apicomplexa, these are members of the superphylum Alveolata, and it has been proposed that orthologs of the well-characterized “Nd” proteins of ciliates comprise one or more portions of the rosette in *Toxoplasma. Plasmodium*, too, has orthologs of these same Nd6 and Nd9 proteins (10, 19, 26, 27), and so it seems highly likely that these serve the same function here.

In both *Plasmodium* merozoites and *Toxoplasma* tachyzoites, freeze-fracture microscopy has revealed the presence of a small, external pit or dimple at the center of the parasite’s apical tip (28, 29). This structure is at the center of the rosette mentioned above and has been speculated to function as part of the channel connecting the parasites’ rhoptries with the host cell cytosol. In this study, we were not able to determine if the rosette might be the source of this surface structure, but the center tip of the rosette lies at about the position of the dimple, so it seems likely that they are related in function, if not physically connected.

In many tomograms, we also detected a small, apical vesicle sandwiched between the rhoptry tip and the rosette, supporting the recently reported observations (10, 30). The location and lower density of this vesicle (relative to the rhoptries) are both reminiscent of the apical vesicles seen in *Toxoplasma* tachyzoites (19, 30, 31). In the latter parasite, the most apical of these vesicles undergoes an apparent fusion with the tip of the rhoptry necks when the parasites are exposed to a calcium ionophore, a stimulus that causes *Toxoplasma’s* conoid to be protruded in preparation for invasion (30). Many have speculated that this apical vesicle is part of the machinery that enables rhoptries to secrete their contents into the host cell through pores at the start of tachyzoite invasion (19), some even referring to such as “porosomes” (31, 32). It is similarly tempting to speculate that the rosette of *Plasmodium* might also serve as a conduit for moving their rhoptry contents into a red blood cell.

Although the precise composition of the apical rings remains unknown, recent studies have localized eight components to this region in *Toxoplasma* tachyzoites (12, 33, 34). Among these eight components, only three (TGME49_201220, TGME49_242320, and TGME49_206430 [15–17]) have orthologs in *Plasmodium* (PlasmoDB.org). Nonetheless, the precise location of these components within the rings is yet to be determined. Given the similarity in the overall shape of the repeating unit within the two rings described here, it seems likely that as more components are described, more will be found homologous between the two genera. Of note, the combined size of the seven proteins so far identified is under 500 KDa, which represents less than 5% of the 13.6–16.3 MDa mass of these structures, indicating that most of them have yet to be identified.

Our subtomogram averaging shows a clear linkage between Ring 1 and Ring 2 with a bridge density connecting the two. This is similar to the overall organization of the preconoidal rings in *Toxoplasma* tachyzoites and as reported for its close relative *Neospora caninum* (35). However, the *Plasmodium* rings had substantially fewer structural units comprising it (34 for *Plasmodium* vs 42–45 for *Toxoplasma* or 45–47 for *Neospora*). In addition, the perturbations in the curvature of the *Plasmodium* rings in some of the tomograms, likely generated during sample preparation, did not break the connection of the repeating ring units, suggesting a resilience to the rings that may play a role in the invasion process when encountering physical barriers within the host cells. It seems likely that the respective compositions of the ring complexes in *Plasmodium* and *Toxoplasma* will be similar, but much work remains to be done on the exact proteins involved and their relative locations within each ring.

## MATERIALS AND METHODS

### *P. falciparum* culture and preparation of free merozoites

*P. falciparum* strain 3D7 was routinely cultured in de-identified human erythrocytes from the Stanford Blood Center at 2% hematocrit at 37°C, 5% CO_2_, and 1% O_2_. The culture medium (termed complete RPMI) consisted of RPMI-1640 (Sigma) supplemented with 25 mM HEPES, 50 mg/L hypoxanthine (Sigma), 2.42 mM sodium bicarbonate, and 4.31 mg/mL Albumax II (Gibco).

Free merozoites were isolated as previously described by Boyle et al. (18), with some modifications. *P. falciparum* was synchronized to the schizont stage using sequential rounds of 5% sorbitol (Sigma) and magnet purification using LS columns and a MACS magnetic separator (Miltenyi Biotec). On the day of harvest, 120 mL of *P. falciparum* culture at 10%–20% parasitemia was subjected to MACS magnet purification to isolate schizonts away from uninfected erythrocytes, yielding ~5 × 10^8^ schizonts. The schizonts were resuspended in 40 mL fresh complete RPMI with 10 µM E-64 (Sigma), a cysteine protease inhibitor that prevents merozoite release from schizonts by inhibiting rupture but does not adversely affect merozoites (18, 36). After a 5-hour incubation at 37°C, the cells were pelleted at 1,500 rpm for 5 minutes, and the supernatant was removed completely. The schizont pellet was resuspended in 6 mL of phosphate-buffered saline (PBS) supplemented with 2% fetal bovine serum (FBS), 5 mM MgCl_2_, and 50 µg/mL DNase I, and was transferred to a syringe connected to a 1.2-µm filter. To release the merozoites, pressure was applied to the syringe plunger evenly until all liquid was expelled. The flow-through was applied to an LS column on a MACS magnet to remove hemozoin, and then centrifuged at 4,000 rpm for 10 minutes, followed by two washes in PBS. The merozoites were quantified using a hemocytometer and resuspended at 5 × 10^7^/mL or 5 × 10^6^/mL.

### *T. gondii* culture and preparation of free tachyzoites

*T. gondii* RH*∆hxgprt* strain was maintained by growth in confluent primary human foreskin fibroblasts (HFFs) in Dulbecco’s modified Eagle’s medium (DMEM; Invitrogen, Carlsbad, CA, USA) with 10% FBS (HyClone, Logan, UT, USA), 2 mM glutamine, 100 U/mL penicillin, and 100 µg/mL streptomycin (cDMEM) at 37°C in 5% CO_2_. The HFFs are fully de-identified and therefore do not constitute human subjects research.

*Toxoplasma* tachyzoites were released from heavily infected monolayers of HFFs by mechanical disruption of the monolayers using disposable scrapers and passage through a syringe attached to a 25-gauge needle. The parasites were added to fresh monolayers of HFFs, and 18–20 hours post-infection, were washed two times with cold Hank’s balanced salt solution (HBSS) without calcium, magnesium, and phenol red (Corning, Corning, NY, USA), supplemented with 1 mM MgCl_2_, 1 mM CaCl_2_, 10 mM NaHCO_3_, and 20 mM HEPES, pH 7. The HFF monolayers were scraped and passed through a 27-gauge syringe, and tachyzoites were released into fresh cold HBSS. The tachyzoites were pelleted and resuspended in fresh cold HBSS, and calcium-ionophore (A23187, Sigma) was added to the sample at room temperature to a final concentration of 1 µM.

### Cryogenic electron tomography

Free merozoites or tachyzoites suspension mixed with 10-nm gold fiducials (EMS) was loaded to the lacey carbon grids, blotted from the back side, and plunged into liquid ethane cooled down to liquid nitrogen temperature using Leica GP. The parasites were imaged using a Talos Arctica electron microscope (Thermo Fisher) equipped with a field emission gun operated at 200 kV, a Volta Phase Plate (37), an energy filter (Gatan) operated at zero-loss, and a K2 Summit direct electron detector (Gatan). Upon phase plate alignment and conditioning, the low-magnification tilt series of whole parasites were recorded at a magnification of 24,000× at pixel size 5.623 Å, and the high-magnification tilt series of the apical region were recorded at a magnification of 39,000× at pixel size 3.543 Å, using Tomo4 software with bidirectional acquisition schemes, each from −60° to 60° with 2- or 3- increment. Target defocus was set to −1.0 µm. The K2 camera was operated in dose fractionation mode recording with 0.4 or 0.5 per e/Å^2^ frame. To increase the contrast, an energy filter was used with a slit width of 20 eV, and a new spot on the phase plate was selected every two or three tilt series. The phase shift spans a range from 0.2 to 0.8π. The total dose was limited to 100–120 e/Å^2^ for merozoite samples and 70–90 e/Å^2^ for tachyzoite samples.

### Tomography reconstruction and analysis

The movie frames were motion-corrected using motionCor2 (38), and the resulting micrographs were compiled into tilt series. For *Plasmodium* merozoites, a total of 18 tomograms of whole merozoites at a magnification of 24,000×, and 39 tomograms at the apical region of merozoites at a magnification of 39,000× were collected. Tilt series alignment, tomography reconstruction, and CTF estimation are performed automatically using the tomography pipeline in EMAN2 (39). For features inspection and presentation, tilt series alignment and reconstruction were performed using IMOD (40, 41). Subcellular features were manually segmented using Fiji or EMAN2 and were viewed in Chimera (42, 43). The repeating units of apical rings were selected and centered manually in the middle of each repeating unit using EMAN2 (39). Subtomogram averages of density maps are then generated with an 80% overlap between neighboring ring units. *De novo* initial models were directly generated from the particles. For the *in situ* apical ring structure of merozoites, 1,156 particles from 39 tomograms were used in the subtomogram average. Starting from the successful subtomogram refinement of the apical ring unit, focused refinement was applied to the bottom part of the ring unit by extracting the particle with a large box size to include Ring 3. For the subtomogram averaging of the apical ring of *Toxoplasma* tachyzoites, 335 particles were generated from 59 tomograms collected with a magnification of 39,000×. The average map of the ring unit can be mapped back into tomograms for assembly of the circular whole rings in *Plasmodium* and *Toxoplasma*. Subsequent visualization and animation of the tomograms and maps were carried out using Chimera or ChimeraX (42).

### Distance measurements

The distance between objects was measured using a line profile plot of the inverted pixel value. The distance between the apicoplast and mitochondria membranes was evaluated along a line crossing at the minimal distance between the organelles. The radii of the *Plasmodium* rings were measured by the outermost density for each of the three rings based on the slice views.

### Map segmentation and alignment

All maps were segmented using the Segger plugin v2.9.1 in UCSF Chimera (44). First, the map of the *Plasmodium* ring was segmented into Ring 1, bridge, and Ring 2. Rings 1 and 2 also contain inner and outer sub-rings (as shown in Fig. S4). The map of the *Plasmodium* apical rings (Fig. 4C) was segmented without using any smoothing and grouping, and the regions were grouped interactively to generate two regions representing Rings 1 and 2 (orange) and Ring 3 (purple). The STA maps (#1, #2, and #3), spanning both Rings 1 and 2, were segmented separately. STA map #1 (Fig. 5A) was segmented using three “smoothing and grouping” steps, then manually joining ~50 regions for each repeating unit interactively. STA maps #2 and #3 (Fig. 5B and C) were segmented similarly. The assembled map (Fig. 5D) was segmented using only one “smoothing and grouping” step, then interactively joining ~10 regions per unit, to correspond to the units segmented in the STA maps. The STA map of the *Toxoplasma* rings (Fig. 6A) was segmented using two “smoothing and grouping” steps, then interactively joining ~15 regions interactively for each repeating unit. The assembled map of the *Toxoplasma* rings was first cropped to ~12 repeating units (Fig. 6C); it was segmented using no “smoothing and grouping,” interactively joining ~10 regions to generate individual repeating units.

For comparison purposes (Fig. 6B), the assembled map of *Toxoplasma* was interactively aligned to the assembled map of *Plasmodium* in UCSF Chimera, and the alignment was refined using the “Fit in Map” dialog. The Volume Tracer tool in Chimera was used to place markers and measure distances between the stalk portions in adjacent repeating units. For Fig. 6C, individual repeating units from *Toxoplasma* and *Plasmodium* maps were also aligned using Chimera, first interactively then using “Fit in Map” for refinement.

The molecular weights of repeating units in each organism (Fig. S6A) were estimated from the volume of each repeating unit measured using the “Measure Volume and Area” dialog in Chimera, assuming an even density throughout the units of 1.35e^−24^ g/A^3^ (45), and the approximate conversion units between kg and Da of 6.022e^23^ Da/g. The spacing between repeating units (Fig. S6B) and the diameter of the *Plasmodium* rings (Fig. S6C) was measured by interactively placing markers on the averaged map using the Volume Tracer tool and “the Distances” dialog in Chimera (42).

## Data Availability

Representative tomograms of *Plasmodium falciparum* and subvolume averages of maps are deposited to EMDB. The accession code for the 3D reconstruction of *Plasmodium* free merozoite is EMD-28142. The accession codes for the reconstructions showing the apical complex of *Plasmodium* are EMD-28138 (side view) and EMD-28141 (top view). The accession codes for the *Plasmodium* averaged maps are EMD-28125 for the whole assembled map of Ring 1 and Ring 2, the subtomogram averaging (STA) map #3 for repeating units of Ring 1, 2, and 3, STA maps #1 and #2 for the repeating unit of Ring 1 and Ring 2. The accession codes for the *Toxoplasma* average map are EMD-28126 for the assembled map of Ring 1 and Ring 2, the STA map for repeating units of Ring 1 and Ring 2.

## SUPPLEMENTAL MATERIAL

Supplemental material mbio.02864-23-s0001.pdf Supplemental figures and video legends.

Video S1 mbio.02864-23-s0002.mp4 Tomogram showing a *Plasmodium falciparum* free merozoite with annotation of key subcellular structures.

Video S2 mbio.02864-23-s0003.mp4 An apical vesicle is located underneath the rosette.

Video S3 mbio.02864-23-s0004.mp4 The rosette can associate directly with the rhoptry neck region.

Video S4 mbio.02864-23-s0005.mp4 The apical polar ring of *Plasmodium* presents a prominent 34-fold symmetry.

Video S5 mbio.02864-23-s0006.mp4 The apical polar rings of *Plasmodium* merozoites and *Toxoplasma* tachyzoites show a similar organization and repeating pattern.

ASM does not own the copyrights to Supplemental Material that may be linked to, or accessed through, an article. The authors have granted ASM a non-exclusive, world-wide license to publish the Supplemental Material files. Please contact the corresponding author directly for reuse.
